# DC/TMD axis I subtyping: generational and gender variations among East Asian TMD patients

**DOI:** 10.1186/s12903-023-03478-x

**Published:** 2023-10-30

**Authors:** Adrian Ujin Yap, Chengge Liu, Jie Lei, Ji Woon Park, Seong Hae Kim, Byeong-min Lee, Kai Yuan Fu

**Affiliations:** 1 Center for TMD & Orofacial Pain, Department of Oral & Maxillofacial Radiology, Peking University School & Hospital of Stomatology, No. 22 Zhong Guan Cun South Ave, Beijing, 100081 China; 2 Department of Dentistry, Ng Teng Fong General Hospital, Faculty of Dentistry, National University Health System, Singapore, Singapore; 3 National Dental Research Institute Singapore, National Dental Centre Singapore and Duke-NUS Medical School, Singapore Health Services, Singapore, Singapore; 4 National Center for Stomatology and National Clinical Research Center for Oral Diseases, Beijing, China; 5 National Engineering Research Center of Oral Biomaterials and Digital Medical Devices, Beijing, China; 6 Department of Oral Medicine, Seoul National University Dental Hospital, Seoul, Korea; 7 Department of Oral Medicine & Oral Diagnosis, Seoul National University School of Dentistry, Seoul, Korea; 8 Dental Research Institute, Seoul National University, Seoul, Korea; 9 Department of Dental Biomaterials Science, Seoul National University School of Dentistry, Seoul, Korea

**Keywords:** Temporomandibular joint disorders, Cohort effect, Gender, Pain, Intra-articular

## Abstract

**Objectives:**

This study examined the generational-gender distinctions in Diagnostic Criteria for Temporomandibular disorders (DC/TMD) subtypes among East Asian patients.

**Methods:**

Consecutive “first-visit” TMD patients presenting at two university-based TMD/orofacial pain clinics in China and South Korea were enlisted. Demographic information along with symptom history was gathered and clinical examinations were performed according to the DC/TMD methodology. Axis I physical diagnoses were rendered with the DC/TMD algorithms and categorized into painful and non-painful TMDs. Patients were categorized into three birth cohorts, specifically Gen X, Y, and Z (born 1965–1980, 1981–1999, and 2000–2012 respectively) and the two genders. Data were evaluated using Chi-square/Kruskal-Wallis plus post-hoc tests and logistic regression analyses (α = 0.05).

**Results:**

Gen X, Y, and Z formed 17.2%, 62.1%, and 20.7% of the 1717 eligible patients examined (mean age 29.7 ± 10.6 years; 75.7% women). Significant differences in prevalences of arthralgia, myalgia, headache (Gen X ≥ Y > Z), and disc displacements (Gen Z > Y > X) were observed among the three generations. Gen Z had substantially fewer pain-related and more intra-articular conditions than the other generations. Women presented a significantly greater frequency of degenerative joint disease and number of intra-articular conditions than men. After controlling for generation-gender interactions, multivariate analyses showed that “being Gen X” and female increased the risk of painful TMDs (OR = 2.20) and reduced the odds of non-painful TMDs (OR = 0.46).

**Conclusions:**

Generational-gender diversities in DC/TMD subtypes exist and are important for guiding TMD care and future research endeavors.

## Background

Temporomandibular disorders (TMDs) are a diverse group of ailments involving the temporomandibular joints (TMJs), masticatory muscles, and their supporting structures. After chronic lower back pain, they are the second most common musculoskeletal condition affecting up to 15% of the adult population [1, 2]. The signs/symptoms of TMDs include jaw joint/muscle pain, headaches, jaw joint sounds, jaw opening, and/or closing difficulties [2]. Correspondingly, common TMDs can be organized into pain-related and intra-articular conditions, as stipulated by the Diagnostic Criteria for TMDs (DC/TMD) standard [2]. The primary subtypes of pain-related TMD conditions (PT) are arthralgia, myalgia, and headache attributed to TMDs, whereas TMJ disc displacement, degenerative joint disease, and subluxation are the primary subtypes of intra-articular TMD conditions (IT). Phenotypic “biopsychosocial” risk factors for TMDs, which are influenced by cultural and environmental milieu, include age, gender, genetics, parafunction activities, co-morbid somatic symptoms/somatization, pain coping/appraisal, and psychological distress [3–7]. The prevalence of TMDs was reported to range from 6 to 16% in the general population [8]. TMD signs/symptoms normally increase during adolescence/young adulthood, peak during middle age, and are low in children and old adults [9–11]. Cross-sectional studies have indicated that women have a higher TMD prevalence and up to 80% of TMD patients are females [8, 11–13] However, “first-onset” TMDs were determined to be positively related to age but weakly associated with gender in a large prospective cohort study [14]. Though “experiences related to aging” were posited as the etiological effect, the phenomenon could arise from “life events” encountered by different generations or birth cohorts.

Interest in generational diversity had increased substantially since the turn of this century [15]. A generation is defined as an identifiable group that shares birth years and significant life events at critical developmental stages [16]. Variances in values, attitudes, and expectations among generations are driven by mutual happenings/collective memories and shaped by cultural heritage [17, 18]. A generation can thus be considered a cohort of persons passing through time who share a “common habitus and lifestyle” [19]. Currently, there are said to be six generational cohorts, namely the “traditionalist”, “baby boomers”, generation X (Gen X), Y (Gen Y) or “millennials”, Z (Gen Z) or “zoomers”, and lastly generation alpha [20, 21]. The distinct traits and “peer personality” of the various generations were described previously [22, 23]. Generations X, Y, and Z are pertinent clinically as they represent the bulk of TMD patients [10, 11].

The worsening mental health of younger generations had been highlighted in several studies [20, 24–26]. A sharp increase in internalizing problems such as depression, anxiety, and somatic symptoms, was observed especially among females [25, 26]. The aforementioned was attributed to rapid and major societal changes in the 21st century including increased urbanization, narcissism, digital connectivity, and deferred life milestones [24–27]. The negative impact of these societal changes on mental health could be greater in East Asians due to latent conflicts with their Confucian traditions [28]. Confucian heritage cultures (CHCs), which consist of countries like China and Korea, emphasize hierarchal harmony, group orientations, interpersonal relationships, and social recognition [29, 30]. The values are underpinned by the pursuit of personal achievements through self-effort and are associated with high levels of psychological distress, a known risk factor for TMDs [6, 7, 29, 31]. Moreover, East Asians are socialized to communicate distress through somatic symptoms including TMDs due to the stigma accompanying mental illness [32–34]. In addition, gender roles and traits may also have some bearing on psychological distress and consequently TMD expression [35].

Information on the frequency of TMD subtypes in patient populations is essential for estimating treatment needs, identifying care priorities, and developing healthcare policies [36, 37]. Generational and gender diversity in TMD subtypes have hitherto not been explored. Therefore, the objective of this study was to establish the generational-gender differences in DC/TMD axis I subtypes among East Asian TMD patients. The research hypotheses were: (a) generational distinctions in TMD subtypes exist, (b) women have more TMD conditions and are particularly susceptible to painful TMDs, and (c) gender variations in the number and frequency of TMD conditions/categories are generation-dependent.

## Methods

### Study design

Data for this study was accrued from a large-scale collaborative investigation of the phenotypic characteristics of East-Asian TMD patients. Ethics approval was granted by the relevant local institutional review boards in China and South Korea (reference: PKUSSIRB-201,732,009 and ERI22001). From 1 Jan 2019 to 31 Dec 2021, data from consecutive “first-visit” patients presenting at two university-based TMD/orofacial pain clinics in Beijing and Seoul were gathered as part of routine diagnostic activities. A minimum sample size of 1,000 subjects per site was fixed a priori to enhance the precision of the prevalence estimates [38]. The inclusion criteria were patients born between 1965 and 2012, Chinese or Korean language proficiency, and the existence of TMD symptoms. Patients with prior orofacial trauma, craniofacial deformities, substance/drug abuse, debilitating psychological, cognitive, or physical impairments, and illiteracy were omitted. At the initial/first visit, demographic information and symptom features were documented using the official Chinese and Korean translations of the DC/TMD Symptom Questionnaire (SQ). The 14-item DC/TMD SQ assesses facial pain, headache, TMJ noises, closed, and open locking in the last 30 days and supplies the necessary history for rendering axis I physical diagnoses. Where applicable, informed consent was obtained for de-identified data usage.

### TMD subtypes and categories

Patients were clinically examined according to the DC/TMD methodology by trained and calibrated oral medicine or TMD/orofacial pain specialists [2]. Items assessed included palpation and movement pain, pain locations, jaw movements/deviations, as well as TMJ noises such as clicking and crepitus. Where indicated, orthopantomography, cone-beam computed tomography (CBCT), and/or magnetic resonance imaging (MRI) were utilized to verify intra-articular disorders. The patients were assigned one or more of the primary axis I physical diagnoses based on the DC/TMD algorithms [2]. The axis I findings were dichotomized into painful (PT without or with IT) and non-painful (IT without PT) TMDs depending on the presence or absence of facial pain. Similarly, TMD illness duration was dichotomized into acute (≤ 3 months) and chronic (> 3 months) for supplementary statistical evaluations [39]. To investigate generational and gender differences in TMD subtypes/categories, patients were stratified into three birth cohorts, specifically Gen X (born 1965–1980), Gen Y (born 1981–1999), and Gen Z (born 2000–2012), and two genders [20].

### Statistical assessment

All statistical assessments were undertaken using the SPSS Statistics software version 27.0 (IBM Corporation, Armonk, New York, USA) with a 5% level of significance (p < 0.05). Frequencies and proportions were used to report qualitative data which were examined using Chi-square/post-hoc Z tests with Bonferroni’s correction. Quantitative data were reported as means/medians with standard deviations (SD)/interquartile ranges. As qualitative data were not normally distributed when examined with the Shapiro-Wilk’s test, they were evaluated using the Kruskal-Wallis/post-hoc Mann-Whitney U tests. Univariate and multivariate logistic regression analyses were performed to determine the generational and gender associations with painful/non-painful TMDs as well as potential interaction effects. Results were reported as odds ratios (ORs) with 95% confidence intervals (95% CIs).

## Results

Of the 2008 “first-visit” TMD patients examined, 1717 met the eligibility criteria. The mean age of the eligible patients was 29.7 ± 10.6 years and 75.7% were women. The distribution of Gen X, Y, and Z was 17.2%, 62.1%, and 20.7% respectively (Table 1). While gender distribution did not differ substantially, significant variances in TMD illness duration were detected (Gen X, Y > Z).

**Table 1:** Demographic characteristics of the East Asian TMD patients

Variables	All patients n (%)	Gen X	Gen Y	Gen Z	P-value *Post-hoc*
Total					
n (%)	1717 (100.0)	295 (17.2)	1067 (62.1)	355 (20.7)	< 0.001* Y > Z,X
**Birth year**	1965–2012	1965–1980	1981–1999	2000–2012	
**Age**					
*Mean (SD)*	29.7 (10.6)	48.0 (4.6)	28.8 (5.0)	17.1 (2.9)	**< 0.001^**
*Median (IQR)*	27.0 (14.0)	49.0 (8.0)	28.0 (7.0)	18.0 (5.0)	X > Y > Z
**Gender**					
*Women, n (%)*	1299 (75.7)	225 (76.3)	818 (76.7)	256 (72.1)	0.216*
*Men, n (%)*	418 (24.3)	70 (23.7)	249 (23.3)	99 (27.9)	
*Female;male (F:M) ratio*	3.1	3.2	3.3	2.6	
**TMD duration (months)**					
*Mean (SD)*	24.4 (48.0)	32.9 (72.6)	26.0 (45.3)	12.9 (19.8)	**0.001^**
*Median (IQR)*	5.5 (23.0)	6.0 (23.0)	6.0 (29.0)	4.0 (17.7)	X,Y > Z

SD = standard deviation; IQR = interquartile range. Results of ^Kruskal-Wallis/Mann-Whitney U tests and *Chi-square/Z tests with Bonferroni correction. Bold indicates p < 0.05

Table 2 shows the frequency of TMD subtypes/categories for the three generations. Arthralgia, myalgia, and headache attributed to TMDs were present in 45.7%, 31.2%, and 9.6% of the patients with significant differences in prevalence among the three generations (arthralgia and myalgia – Gen X > Y > Z; headache – Gen X, Y > Z). Gen X had significantly more pain conditions than Gen Y and Z (Gen X > Y > Z). TMJ disc displacements, degenerative joint disease, and subluxation were present in 76.8%, 37.5%, and 1.5% of the patients with significant differences in the prevalence of disc displacements among generations (Gen Z > Y > X). Gen Z had significantly more intra-articular conditions than the other two generations (Gen Z > Y, X). Substantial variations in the total number of TMD conditions and prevalence of painful TMDs were also observed (Gen X > Y > Z).

**Table 2:** Frequency of TMD subtypes/categories for the three generations

Variables	All patients n (%)	Gen X	Gen Y	Gen Z	P-value *Post-hoc*
Total					
n (%)	1717 (100.0)	295 (17.2)	1067 (62.1)	355 (20.7)	< 0.001* Y > X,Z
**Pain-related TMD conditions (PT)**					
*Arthralgia*	784 (45.7)	172 (58.3)	484 (45.4)	128 (36.1)	< 0.001* X > Y > Z
*Myalgia*	536 (31.2)	137 (46.4)	343 (32.1)	56 (15.8)	< 0.001* X > Y > Z
*Headache*	165 (9.6)	37 (12.5)	118 (11.1)	10 (2.8)	< 0.001* X,Y > Z
**Number of pain-related conditions**					
*Mean (SD)*	0.86 (0.84)	1.17 (0.80)	0.89 (0.86)	0.55 (0.70)	**< 0.001^** X > Y > Z
*Median (IQR)*	1.0 (2.0)	1.0 (1.0)	1.0 (1.0)	0 (1.0)	
**Intra-articular TMD conditions (IT)**					
*Disc displacements (DD)*	1318 (76.8)	202 (68.5)	811 (76.0)	305 (85.9)	< 0.001* Z > Y > X
*Degenerative joint disease*	644 (37.5)	109 (36.9)	394 (36.9)	141 (39.7)	0.627*
Subluxation	25 (1.5)	2 (0.7)	19 (1.8)	4 (1.1)	0.276*
**Number of intra-articular conditions**					
*Mean (SD)*	1.16 (0.59)	1.06 (0.67)	1.15 (0.58)	1.27 (0.53)	**< 0.001^** Z > Y,X
*Median (IQR)*	1.0 (1.0)	1.0 (0)	1.0 (0)	1.0 (1.0)	
**Total number of TMD conditions**					
*Mean (SD)*	2.02 (1.01)	2.23 (1.03)	2.03 (1.02)	1.81 (0.89)	**< 0.001^** X > Y > Z
*Median (IQR)*	2.0 (2.0)	2.0 (2.0)	2.0 (2.0)	2.0 (1.0)	
**TMD categories**					
*Painful TMDs*	1051 (61.2)	240 (81.4)	656 (61.5)	155 (43.7)	< 0.001* X > Y > Z Z > Y > X
*Non-painful TMDs*	666 (38.8)	55 (18.6)	411 (38.5)	200 (56.3)	
**TMD duration**					
*Acute (≤ 3 months)*	713 (41.5)	114 (38.6)	448 (42.0)	151 (42.5)	0.535*
*Chronic (> 3 months)*	1004 (58.5)	181 (61.4)	619 (58.0)	204 (57.5)	
*Acute:chronic (A:C) ratio*	0.71	0.63	0.72	0.74	

SD = standard deviation; IQR = interquartile range. Results of ^Kruskal-Wallis/Mann-Whitney U tests and *Chi-square/Z tests with Bonferroni correction. Bold indicates p < 0.05

Table 3 reflects the frequency of TMD subtypes/categories for the two genders. No significant differences in the prevalence of arthralgia, myalgia, headache, and the number of pain-related TMD conditions were discerned between genders. Besides TMJ degenerative joint disease, no significant differences in the prevalence of intra-articular conditions were also noted. Women had a substantially greater prevalence of degenerative joint disease than men (40.3% versus 28.7%). They also presented significantly more intra-articular conditions and a greater total number of TMD conditions. The variations in frequency of painful/non-painful and acute/chronic TMDs were insignificant between genders.

**Table 3:** Frequency of TMD subtypes/categories for the two genders

Variables	All patients n (%)	Female (F)	Male (M)	P-value *Post-hoc*
Total				
n (%)	1717 (100.0)	1299 (75.7)	418 (24.3)	< 0.001* F > M
**Pain-related TMD conditions (PT)**				
*Arthralgia*	784 (45.7)	608 (46.8)	176 (42.1)	0.093*
*Myalgia*	536 (31.2)	411 (31.6)	125 (29.9)	0.505*
*Headache*	165 (9.6)	132 (10.2)	33 (7.9)	0.171*
**Number of pain-related conditions**				
*Mean (SD)*	0.86 (0.84)	0.89 (0.86)	0.80 (0.79)	0.110^
*Median (IQR)*	1.0 (2.0)	1.0 (1.0)	1.0 (1.0)	
**Intra-articular TMD conditions (IT)**				
*Disc displacements (DD)*	1318 (76.8)	1007 (77.5)	311 (74.4)	0.189*
*Degenerative joint disease*	644 (37.5)	524 (40.3)	120 (28.7)	< 0.001* F > M
Subluxation	25 (1.5)	19 (1.5)	6 (1.4)	0.968*
**Number of intra-articular conditions**				
*Mean (SD)*	1.16 (0.59)	1.19 (0.58)	1.05 (0.59)	**< 0.001^** F > M
*Median (IQR)*	1.0 (1.0)	1.0 (1.0)	1.0 (0)	
**Total number of TMD conditions**				
*Mean (SD)*	2.02 (1.01)	2.08 (1.02)	1.84 (0.94)	**< 0.001^** F > M
*Median (IQR)*	2.0 (2.0)	2.0 (2.0)	2.0 (1.0)	
**TMD categories**				
*Painful TMDs*	1051 (61.2)	888 (68.4)	248 (59.3)	0.282*
*Non-painful TMDs*	666 (38.8)	411 (31.6)	170 (40.7)	
**TMD duration**				
*Acute (≤ 3 months)*	713 (41.5)	530 (40.8)	183 (43.8)	0.364*
*Chronic (> 3 months)*	1004 (58.5)	769 (59.2)	235 (56.2)	
*Acute:chronic (A:C) ratio*	0.71	0.69	0.78	

Results of ^Mann-Whitney U tests and *Chi-square tests. Bold indicates p < 0.05

Gender differences in the number and frequency of TMD conditions/categories for the three generations are reflected in Table 4. A predominance of women was observed for all three generations and the proportion of female-to-male patients did not vary much. Unlike Gen Y and Z, Gen X women had significantly more pain-related TMD conditions than their male counterparts. For both genders, substantial variations in the number of pain-related conditions were discerned among generations (female – Gen X > Y > Z; male – Gen X, Y > Z). For all three generations, women had significantly more intra-articular conditions. Substantial variations in the number of intra-articular conditions were again noted among generations (female – Gen Z > Y, X; male – Gen Z > X). Regarding the total number of TMD conditions, Gen X and Y women had significantly more TMD conditions than men. Substantial differences in the total number of TMD conditions among generations were observed only in women (Gen X > Y > Z). While gender differences in the prevalence of painful and non-painful TMDs were noted for Gen X, the variances were insignificant for Gen Y and Z. For the two genders, generational differences in prevalence were inverted (painful TMDs – Gen X > Y > Z; non-painful TMDs - Gen Z > Y > X). However, generational differences in TMD illness duration varied significantly only in women (Gen X, Y > Z).

**Table 4:** Gender differences in the number and frequency of TMD conditions/categories for the three generations

Generation	Variables	Female (F)	Male (M)	P-value
	Total			
Gen X	295 (17.2)	225 (76.3)	70 (23.7)	< 0.001*, F > M
Gen Y	1067 (62.1)	818 (76.7)	249 (23.3)	< 0.001*, F > M
Gen Z	355 (20.7)	256 (72.1)	99 (27.9)	< 0.001*, F > M
	***Column P-value***	0.216*		
	**Number of pain-related conditions**			
Gen X	*Mean (SD)*	1.25 (0.81)	0.93 (0.73)	**0.005^** F > M
	*Median (IQR)*	1.0 (1.0)	1.0 (1.0)	
Gen Y	*Mean (SD)*	0.89 (0.87)	0.88 (0.84)	0.960^
	*Median (IQR)*	1.0 (1.0)	1.0 (1.0)	
Gen Z	*Mean (SD)*	0.56 (0.71)	0.52 (0.66)	0.731^
	*Median (IQR)*	0 (1.0)	0 (1.0)	
	***Column P-value*** *Post-hoc*	**< 0.001^** X > Y > Z	**< 0.001^** X,Y > Z	
	**Number of intra-articular conditions**			
Gen X	*Mean (SD)*	1.12 (0.67)	0.89 (0.63)	**0.012^** F > M
	*Median (IQR)*	1.0 (1.0)	1.0 (1.0)	
Gen Y	*Mean (SD)*	1.18 (0.57)	1.04 (0.61)	**0.001^** F > M
	*Median (IQR)*	1.0 (1.0)	1.0 (0)	
Gen Z	*Mean (SD)*	1.30 (0.53)	1.17 (0.50)	**0.034^** F > M
	*Median (IQR)*	1.0 (1.0)	1.0 (0)	
	***Column P-value*** *Post-hoc*	**0.003^** Z > Y,X	**0.008^** Z > X	
	**Total number of TMD conditions**			
Gen X	*Mean (SD)*	2.36 (1.04)	1.81 (0.89)	**< 0.001^** F > M
	*Median (IQR)*	2.0 (1.0)	2.0 (1.0)	
Gen Y	*Mean (SD)*	2.07 (1.04)	1.92 (0.97)	**0.048^** F > M
	*Median (IQR)*	2.0 (2.0)	2.0 (2.0)	
Gen Z	*Mean (SD)*	1.86 (0.90)	1.69 (0.86)	0.070^
	*Median (IQR)*	2.0 (1.0)	1.0 (1.0)	
	***Column P-value*** *Post-hoc*	**< 0.001^** X > Y > Z	0.139^	
	**Painful TMDs**			
Gen X	240 (22.8)	190 / 225 (84.4)	50 / 70 (71.4)	0.015* F > M
Gen Y	656 (62.4)	501 / 818 (61.2)	155 / 249 (62.2)	0.776*
Gen Z	155 (14.7)	112 /256 (43.8)	43 / 99 (43.4)	0.957*
	***Column P-value***	< 0.001* X > Y > Z	< 0.001* X > Y > Z	
	**Non-painful TMDs**			
Gen X	55 (8.3)	35 / 225 (15.6)	20 / 70 (28.6)	0.015* M > F
Gen Y	411 (61.7)	317 / 818 (38.8)	94 / 249 (37.8)	0.776*
Gen Z	200 (30.0)	144 / 256 (56.3)	56 / 99 (56.6)	0.957*
	***Column P-value***	< 0.001* Z > Y > X	< 0.001* Z > Y > X	
	**TMD duration (months)**			
Gen X	*Mean (SD)*	32.6 (72.35)	33.9 (73.75)	0.190^
	*Median (IQR)*	6.0 (22.5)	3.0 (18.5)	
Gen Y	*Mean (SD)*	25.0 (45.21)	29.0 (45.67)	0.587^
	*Median (IQR)*	6.0 (23.0)	5.0 (36.0)	
Gen Z	*Mean (SD)*	11.8 (19.11)	15.5 (21.33)	0.036^
	*Median (IQR)*	3.0 (11.9)	6.0 (23.0)	
	**Column P-value** *Post-hoc*	**< 0.001^** X,Y > Z	0.900^	

SD = standard deviation; IQR = interquartile range. Results of ^Kruskal-Wallis/Mann-Whitney U tests and *Chi-square/Z tests with Bonferroni correction. Bold indicates p < 0.05

Table 5 presents the outcomes of univariate and multivariate regression analyses with interaction effects. With the univariate modeling, both painful and non-painful TMDs were associated with generation and TMD illness duration but not gender. Multivariate analyses indicated that the odds of painful TMDs were increased by “being Gen X (OR = 3.02; 95% CI = 1.72–5.30) and Gen Y (OR = 2.00; 95% CI = 1.43–2.79)”. When generation-gender interaction effects were factored the odds of painful TMDs were reduced substantially for Gen X (OR = 2.20; 95% CI = 1.16–4.14) and Gen Y (OR = 0.98; 95% CI = 0.73–1.31) women. Conversely, a protective effect against non-painful TMDs was observed by “being Gen X (OR = 0.33; 95% CI = 0.19–0.58) and Gen Y (OR = 0.50; 95% CI = 0.36–0.70)”. Again the odds for Gen X (OR = 0.46; 95% CI = 0.24–0.86) and Gen Y (OR = 1.00; 95% CI = 0.99-1.00) women were moderated by generation-gender interactions. Though TMD illness duration was significantly related to the presence of both painful and non-painful TMDs, ORs were equal to 1.

**Table 5:** Results of univariate and multivariate analyses

	Univariate		Multivariate	
Variables	Odds ratio (95% CI)	P-value*	Odds ratio (95% CI)	P-value^
Painful TMDs				
**Gender**				
*Women*	1.11 (0.89–1.39)	0.364		
*Men*	Reference			
**Generation**				
*X*	5.63 (3.93–8.07)	**< 0.001**	3.02 (1.72–5.30)	**< 0.001**
*Y*	2.06 (1.62–2.63)	**< 0.001**	2.00 (1.43–2.79)	**< 0.001**
*Z*	Reference		Reference	
**TMD duration**	1.01 (1.00-1.01)	**< 0.001**	1.00 (1.00-1.01)	**0.001**
Generation*Gender				
X*Female			2.20 (1.16–4.14)	**0.015**
Y*Female			0.98 (0.73–1.31)	0.863
Z*Male			Reference	
**Non-painful TMDs**				
**Gender**				
*Women*	0.90 (0.72–1.13)	0.364		
*Men*	Reference			
**Generation**				
*X*	0.18 (0.12–0.26)	**< 0.001**	0.33 (0.19–0.58)	**< 0.001**
*Y*	0.49 (0.38–0.62)	**< 0.001**	0.50 (0.36–0.70)	**< 0.001**
*Z*	Reference		Reference	
**TMD duration**	1.00 (0.99-1.00)	**< 0.001**	1.00 (0.99-1.00)	**0.001**
Generation*Gender				
X*Female			0.46 (0.24–0.86)	**0.015**
Y*Female			1.00 (0.99-1.00)	0.863
Z*Male			Reference	

*Results of univariate and multivariate logistic regression analyses. Bold indicates p < 0.05*

## Discussion

This study is the first to explore the generational and gender differences in DC/TMD axis I subtypes and serves as a resource for similar work in other cultures. As the prevalence of TMD subtypes differed between the three generations and gender variances in the number/frequency of TMD conditions/categories were generation-dependent, the first and third hypotheses were endorsed. The second hypothesis was only partly supported as women had more TMD conditions but were similarly susceptible to painful TMDs when compared to men. The global adoption and systematic translation of the DC/TMD and its antecedent, the Research Diagnostic Criteria for TMDs (RDC/TMD), have facilitated data coalition and comparison across countries. Manfredini et al., in their meta-analysis of 3,463 TMD patients, specified a female-to-male (F:M) ratio of 3.3 and overall prevalences of 45.3% for myalgia, 41.1% for disc displacements, and 30.1% for TMJ arthralgia/degenerative joint disease based on the RDC/TMD [8]. Though the F:M ratio of East Asian TMD patients (3.1) was comparable, dissimilarities in the frequencies of myalgia (31.2%), disc displacements (76.8%), and TMJ arthralgia/degenerative joint disease (45.7%/37.5%) were discerned. Apart from racial differences, the inconsistencies could also reflect variations in eligibility criteria, TMD definitions/groupings, and methodology employed. Gen X, Y, and Z formed 85.5% of all “first-visit” TMD patients with the “millennials” comprising the majority of eligible patients. The latter was not surprising as Gen Y constituted the largest proportion of the workforce and were found to have poorer health, more chronic conditions, and moderate-to-severe psychological distress (which are associated with TMDs) than their preceding generation [5–7, 24, 40]. Furthermore, “millennials” also experience higher levels of negative emotions, worry, and rumination than older generations when exposed to significant “life events” such as the Covid-19 pandemic [41]. The three most common TMD subtypes encountered in Gen Y were all joint-related (TMJ disorders), namely disc displacements (76.0%), arthralgia (45.4%), and degenerative joint disease (36.9%).

### Generational difference in TMD subtypes/categories

The “birth cohort” comparison approach employed offered a unique framework for examining generational differences in illness expression, health beliefs, help-seeking behaviors, and treatment decision-making as individuals from each cohort were reckoned to have similar characteristics [42]. Generational variations in the type, number, and duration of TMD conditions were detected. Gen X and Y reported substantially longer durations of TMD illness (mean of 26.0 to 32.9 months) at their initial visit when contrasted to Gen Z (mean of 12.9 months), suggesting delayed or deferred help-seeking behaviors. This might be attributed to the self-reliant/cynical nature of Gen X and the optimistic/tolerant disposition of Gen Y [22, 23]. While Gen X and Y presented substantially higher prevalence and number of pain-related conditions than Gen Z (Gen X ≥ Y > Z), the converse was true of disc displacements and the number of intra-articular conditions (Gen Z > Y ≥ X). Although findings can be explained by purported age-related experiential changes such as workplace stressors, generational differences in general/mental health, emotional responses, pain beliefs, and attitudes may also play a part [14, 40, 41, 43, 44]. Younger generations were found to agree more with current pain neuroscience and accepted pain as “normal and part of the survival mechanism” and that its presence does not indicate “something wrong with one’s tissues” [44]. Gen Z, being digital natives, are known to process the latest information faster than any other generations. While TMD pain is the usual reason for TMD treatment-seeking, Gen Z patients sought help mostly for TMD dysfunction (mainly disc displacements) and had lower occurrences of arthralgia, myalgia, and headache than Gen Y and X [1]. Considerable differences in the number of pain-related conditions and frequency of painful TMDs were also observed (Gen X > Y > Z). Findings corroborated those of other clinical investigations alluding to an increase in pain prevalence with advancing age. However, the results of experimental investigations were ambivalent with studies indicating both increased and decreased pain thresholds with age [45].

### Gender difference in TMD subtypes/categories

Three-quarters of the eligible TMD patients were women and they had a significantly greater total number of TMD conditions than men. Findings were consistent with the higher risk of TMDs in women which was attributed to gender disparities in biology, psychological distress, social functioning, pain threshold/tolerance, and help-seeking behaviors [12, 13, 46]. Though it has been reported that women have more severe and frequent pain than men, no significant differences in the prevalence and number of pain-related TMD conditions were noted between genders [46]. This phenomenon could be rationalized by the help-seeking behaviors of male East-Asian TMD patients who appear to be pursuing professional treatment largely for painful TMDs [47]. Despite a similar frequency of disc displacements, women had a 1.4 folds greater prevalence of TMJ degenerative joint disease than men. Fluctuating levels of female sex hormones during puberty, pregnancy, and menopause had been implicated in both TMD pain and TMJ degeneration [12, 13]. However, the underlying mechanisms remain unclear with estrogen playing a possible destructive role in the condylar cartilage but a protective one in the subchondral bone [48]. This could also clarify the high frequencies of TMJ disorders among Gen Y patients who are mostly women.

### Generation and gender interaction

Gender variations in the number of pain-related and total TMD conditions changed depending on generation. Likewise, generational variations in the number of intra-articular, total TMD conditions, and TMD duration were detected between women and men. Additionally, significant gender variations in the prevalences of painful and non-painful TMDs were only observed for Gen X. Therefore, an interaction effect where gender may have a different consequence on TMD outcomes depending on generation and contrariwise might be present. The joint or synergistic effect could be significantly greater or lesser than generation or gender acting in isolation and necessitates examination in the multivariate regression model. Univariate analysis indicated that painful and non-painful TMDs were related to generation and TMD illness duration but not gender. After controlling for generation-gender interaction effects in the multivariate model, the risk of painful TMDs was doubled whereas that of non-painful TMDs was halved by being Gen X and female. Though illness duration was significantly associated with painful and non-painful TMDs, ORs were equal to 1 indicating weak or no affiliations. This could be qualified by the high precision (narrow 95% CI) and large sample size achieved with this study [49]. With large sample sizes, the distribution function of the OR tends to converge to a normal distribution centered on the estimated effect. Given the preponderance of women among TMD patients, the greater prospect of Gen X experiencing TMD pain, and younger generations having TMD dysfunction, generational-gender diversities must be considered when formulating TMD care and healthcare policies as well as directing future TMD research. The latter could entail generational-gender effects on patient beliefs, treatment-seeking behaviors, expectations, and decision-making concerning TMDs. Public health initiatives focusing on the mind-body wellness of younger generations, particularly the “millennials”, should also be introduced because of the increased psychological distress and somatic symptoms including TMDs encountered [25, 26]. While conservative management of TMDs typically includes a combination of patient education/self-management, psychological, pharmacological, physical, and occlusal appliance therapy, interventions for central sensitization syndromes such as antidepressants, cognitive behavior, and mindfulness therapy, could also be beneficial for TMDs, owing to the mind-body connections [50, 51].

### Study limitations

Generational health research is still in its infancy and this study has its limitations.

First, a cross-sectional design instead of a longitudinal one was applied. While this study yielded valuable information, greater generational insights could be attained by the prospective evaluation of the different generations at the same age (for example generation X, Y, and Z at 25 years of age). However, this research would require about 30 to 40 years to be actualized as each generation spans over 12 to 15 years. Second, only East Asian TMD patients were examined. As some racial and cultural distinctions are foreseen, this study must be repeated in Western and other Asian countries to confirm the present findings. The study could also be extended to other birth cohorts such as “generation alpha” and the “baby boomers”, despite their relatively small numbers, with consideration of explicit physical and psychosocial changes in these generations. Third, the stratification of birth cohorts was based on the American standard and may not be completely applicable to East Asian populations due to variances in political, societal, economic, or technological developments. Nevertheless, the findings serve as an initial step in the study of generational diversity in TMDs. Lastly, just generational-gender variances in physical TMD subtypes were delved into. Follow-up work could incorporate psychosocial and behavioral assessments of the different birth cohorts.

## Conclusion

The vast majority of patients seeking TMD treatment are “millennials” and women.

Significant differences in prevalences of arthralgia, myalgia, headache attributed to TMDs (Gen X ≥ Y > Z), and disc displacements (Gen Z > Y > X) were discerned among the three generations. Furthermore, Gen Z had substantially fewer pain-related and more intra-articular conditions than the other generations. Women presented a significantly greater frequency of degenerative joint disease and number of intra-articular conditions than men. Generational-gender interaction effects were evident. Being Gen X and female doubled the risk of painful TMDs but halved the prospect of non-painful TMDs. Though age-related experiential changes may be responsible, generational disparities in societal stress, general/mental health, emotional response as well as pain attitudes, beliefs, and help-seeking behaviors may play crucial roles. Generational-gender diversities must be considered when formulating TMD care and healthcare policies as well as future TMD research.

## Data Availability

The datasets generated and analysed during the current study are available from the corresponding author on reasonable request.

## List of abbreviations

DC/TMD: Diagnostic criteria for Temporomandibular disorder
Gen X, Y, and Z: Generation X, Y, and Z
IT: Intra-articular Temporomandibular disorders
PT: Pain-related Temporomandibular disorders
TMDs: Temporomandibular disorders
